# The relationship between anxiety, enjoyment, and breakdown fluency during second language speaking tasks: An idiodynamic investigation

**DOI:** 10.3389/fpsyg.2022.968946

**Published:** 2022-08-30

**Authors:** Scott Aubrey

**Affiliations:** Department of Curriculum and Instruction, Faculty of Education, The Chinese University of Hong Kong, Hong Kong, Hong Kong SAR, China

**Keywords:** anxiety, enjoyment, fluency, task-based language teaching, idiodynamic method, second language learning, spoken task performances

## Abstract

Research has found that levels of enjoyment and anxiety fluctuate on a moment-to-moment timescale during second language (L2) spoken task performances as learners attempt to cope with various communication challenges. For L2 speakers, surges in these emotions can limit or expand cognitive resources, affecting speech processing capability. However, at an intra-individual level, there is very little empirical evidence on how emotions and fluency are related during L2 spoken task performances. The present study uses the idiodynamic approach to examine the relationship between enjoyment, anxiety, and breakdown fluency (i.e., average length of pauses) during monolog tasks performed by university students who use English as an L2. After watching a video recording of their task performances, participants rated their anxiety and emotion levels on a per-second basis. Immediately after, they were interviewed about their attributions for fluctuations in their ratings. After segmenting task performances into 26 7-s segments of speech, per-person correlations revealed that (1) the (negative) relationship between anxiety and enjoyment varied from strong to very weak, and (2) the (positive) relationship between anxiety and breakdown fluency was much stronger than the (negative) relationship between enjoyment and breakdown fluency. Triangulation of anxiety and enjoyment ratings, stimulated recall interviews, and performance data led to the identification of four categories of factors that influenced the emotion-fluency relationship: task design factors (task structure, task topic), task implementation factors (pre-task planning, task time limits), cognitive-linguistic factors (momentary breakdowns in conceptualization and formulation speech processes), and achievement outcome factors (self-evaluations of appropriate and inappropriate language used).

## Introduction

With the so-called “affective turn” in second language acquisition (SLA) (Pavlenko, 2013), there has been a growing understanding that emotions play an important role in second language (L2) learning (e.g., Swain, 2013; Prior, 2019; Lambert and Zhang, 2019; MacIntyre and Wang, 2021). Although the exact mechanism is unclear, scholars agree that emotions tend to motivate and influence cognition (Izard, 2009). Specifically, negative emotions are thought to restrict learners’ attention and cognitive resources, whereas positive emotions play a facilitative function by broadening learners’ perspectives (Fredrickson, 2004). Given that emotions can fluctuate rapidly during language production (Boudreau et al., 2018), the cognitive processes that impact speech fluency during communication may also undergo temporary changes.

The role that emotion and cognition play in speech production can be interpreted through Levelt’s (1989, 1999) model of first language speech production, which divides the speech production process into three sequential stages: *conceptualization*, when the speaker decides the relevant content to use; *formulation*, when the speaker encodes the content with relevant lexis and grammar in preparation for expression; and *articulation*, where a phonological plan is mapped onto the formulated message. In the case of L2 learners, disfluent speech is related to their inability to process L2 speech efficiently in real-time communication (Kormos, 2006; Skehan, 2014). Thus, momentary fluctuations in emotions may cause momentary disturbances in cognitive processing at the conceptualization or formulation stage, enabling more or less fluent speech at times throughout a performance. However, researching the dynamic role emotion plays in speech production during tasks requires innovative methods to capture the within-person variability of emotion at a micro-level. Such methods are beyond the prevalent “post-task questionnaire” approach used in task-based language teaching (TBLT) research.

To address this issue, the current study uses the idiodynamic approach (MacIntyre and Ducker, 2022) to examine the relationship between anxiety, enjoyment and breakdown fluency (average length of pausing) during monolog L2 speaking tasks. This relationship is explored by dividing participants’ monolog task performances into short, segmented units of speech, establishing per-person correlations between the two emotion variables and breakdown fluency, and exploring reasons behind momentary or prolonged changes in enjoyment and anxiety during task performances. This research is potentially important as it establishes an agenda for investigating emotions and language production in TBLT research from a complex dynamics systems theory (CDST) perspective.

## Literature review

### Emotions in second language acquisition

Emotion can be described as consisting of “neural circuits (that are at least partially dedicated), response systems, and a feeling state/process that motivates and organizes cognition and action” (Izard, 2010, p. 367). SLA researchers have gone so far as to suggest that emotion and cognition are “inseparable” (Swain, 2013). At the very least, emotions are thought to direct cognitive resources, affecting attention, memory, and behavior (Schumann, 1994; Pekrun, 2006). A spectrum of emotions has been identified as influencing language learning and communication. These include *positive emotions* such as enjoyment, love, pride, or gratitude, and *negative* emotions such as anxiety, boredom, guilt, and anger (for a review, see Shao et al., 2020). The characteristics of emotions can be understood through the multidimensional model of affect (Linnenbrink, 2007), which describes emotions as having dimensions of valence (e.g., *love*: negative; *guilt*: positive) and activation (e.g., *anxiety*: high activation; *boredom*: low activation). An implication of this model is that both negative and positive emotions can be highly activating (e.g., *anxiety, enjoyment*), energizing learners in terms of motivation, effort, and performance. From another perspective, the control-value theory framework (Pekrun, 2006) posits that emotions can be conceptualized in terms of achievement. That is, they can be either connected to achievement activities (e.g., frustration from a difficult task), with resultant *anticipatory emotions* (e.g., hopelessness) or connected to achievement outcomes (e.g., anxiety of failure at task), with resultant *retrospective emotions* (e.g., shame). Such classifications are important in SLA as they provide frameworks for hypothesizing which emotions facilitate or inhibit learning behavior as well as how emotions are generated during learning tasks.

Of the multitude of emotions that language learners can experience, by far the most researched emotion in SLA is anxiety, defined as “the worry and negative emotional reaction aroused when learning or using a second language” (MacIntyre, 1999, p. 27). High levels of anxiety can be debilitating for language performance and hinder language acquisition (Horwitz, 2010; Gregersen and MacIntyre, 2014) as it can lead to disruptions in cognitive ability and cause one to withdrawal from involvement to seek self-protection (Arnold and Brown, 1999). However, as a high activation emotion, anxiety has also been shown to motivate learners, causing them to increase effort in the face of increased cognitive demands (Marcos-Llinaìs and Garau, 2009).

Recent interest in positive psychology in SLA (MacIntyre and Gregersen, 2012; MacIntyre and Mercer, 2014) has resulted in an uptick in research investigating the role of positive emotions in language learning, particularly enjoyment. Enjoyment can be broadly defined as an experience, event, or action that is perceived as offering joy, happiness, and pleasure (Brantmeier, 2005). Enjoyment is also used synonymously with *flow*, or “a deep, spontaneous involvement with the task at hand” (Csikszentmihalyi, 2014, p. 181). In a similar way, Dewaele and MacIntyre (2016) define enjoyment as “a complex emotion, capturing interacting dimensions of the challenge and perceived ability that reflects the human drive for success in the face of difficult tasks” (p. 216). Scholars, such as Csikszentmihalyi and Dewaele, thus emphasize the productive aspect of enjoyment, highlighting that it is a positive, activity-focused emotion.

The relationship between enjoyment and anxiety has been the target of recent interest in SLA (e.g., Dewaele and MacIntyre, 2014; Boudreau et al., 2018; Dewaele and Pavelescu, 2021). Rather than comprising opposite ends of the same dimension, current thinking suggests enjoyment and anxiety are independent emotions (Dewaele and MacIntyre, 2014). As enjoyment and anxiety are highly activating, they both serve to energize learners’ behaviors in complementary ways (Linnenbrink, 2007). Supporting this view, Elahi Shirvan and Talebzadeh (2018a) found that, in the same L2 learning environment, learners experienced both anxiety, as a result of pressure from time limits or competitive goals, and enjoyment, caused by positive feedback or a sense of achievement. Both emotions “pushed [them] forward to take actions in the process of learning” (p. 127). Thus, an important emerging viewpoint is that enjoyment and anxiety do not always change together in a “see-saw fashion” (Dewaele and Pavelescu, 2021) but converge and diverge over time (Dewaele and MacIntyre, 2014). This nuanced perspective suggests that anxiety and enjoyment can be co-occurring emotions, and successful language learners must “embrace ‘joy’ as well as ‘pain”’ (Prior, 2019, p. 522).

### Anxiety and enjoyment in spoken L2 tasks

One way to deepen our understanding of how anxiety and enjoyment emerge in language learning activities, their relationship to each other, and their impact on cognition, is through examining learners’ performances during L2 tasks. In SLA, the study of emotion in L2 speaking has been researched from the perspective of both TBLT and L2 psychology.

TBLT research has a pedagogic focus and views tasks as distinct units of learning that create meaningful communication situations where the L2 can be learned *through* language use (Ellis et al., 2020). Recent research in this vein seeks to understand how task design and implementation conditions can affect learners’ emotional responses in relation to task performance. A consistent finding is that designing tasks with familiar content can engender greater levels of interest, enthusiasm, confidence, and enjoyment (Aubrey, 2017a,b, 2022; Lambert et al., 2017, 2022; Qiu and Lo, 2017; Lambert and Zhang, 2019; Aubrey et al., 2020; Nakamura et al., 2021). Such tasks have an embedded dimension of learner control (e.g., students choose their own photo in a photo-description task) and so provide learners with a sense of meaning and autonomy—a key pre-condition for the emergence of positive emotions (Csikszentmihalyi et al., 2005; Aubrey, 2017b,^2021^). However, the effect of learner control on both enjoyment and anxiety can be complex. For example, Nakamura et al. (2021) found that giving learners such control—in their case, choice over task content—led to increases in anxiety *and* enjoyment. This suggests that when learners are free to use their own ideas and language during a task, they may enter into a vulnerable, anxious state, but this state is necessary for the kind of exploration and play that generates feelings of enjoyment. Certain task implementation factors, particularly, pre-task planning, can also affect learners’ task emotions (e.g., Kim, 2013; Bui and Teng, 2018). As planning can reduce the cognitive burden of conceptualization and formulation (Skehan, 2018), planning content and language to be used may lead to overall reduced anxiety during performances. However, as Bui and Teng (2018) argue, some learners prefer not to plan because preparation constrains learners’ choices and limits their ability to speak extemporaneously on a topic. In a similar way, repeating a task may alleviate anxiety as learners need to devote progressively less attention to lexical retrieval processes with each attempt (Bygate and Samuda, 2005), but too many repetitions of the same task can cause boredom/apathy as they become “tired of doing the same thing” (Kim, 2013, p. 17).

Despite the aforementioned insights, methodologically, TBLT research has been mostly limited to using retrospective post-task questionnaires to measure emotional experiences. Thus, task-based research has largely ignored the substantial within-person variation in emotion levels that occur *during* a task. Filling this gap, researchers in L2 psychology have developed new methods which aim to describe “the complex intraindividual emotional reactions and changes in self-perception, not group averages, that occur during brief episodes… of L2 communication” (MacIntyre and Ducker, 2022, p. 1). These studies employ the idiodynamic method, which involves recording an episode of task communication, having participants repeatedly rate affective states on a per-second basis using computer software while watching their recorded performance, and conducting a stimulated recall with participants to elicit reasons for changes in ratings. While idiodynamic studies often measure conative variables, such as willingness to communicate (WTC) (e.g., MacIntyre and Legatto, 2011; Ducker, 2021), others have measured dynamic changes in emotions, such as anxiety (Gregersen et al., 2014; Elahi Shirvan and Talebzadeh, 2018b; MacIntyre, 2019; Macintyre and Gregersen, 2022), enjoyment (Talebzadeh et al., 2020) or both anxiety and enjoyment (Boudreau et al., 2018). These studies take a CDST perspective (Larsen-Freeman and Cameron, 2008), demonstrating that emotions fluctuate during communication as learners attempt to cope with ongoing communication demands.

Most idiodynamic studies have employed dyadic tasks (conversations, interviews). A common finding is that changes in emotions tend to be related to interpersonal issues. For example, Elahi Shirvan and Talebzadeh (2018b) found that learners’ anxiety levels faced constant change, which was influenced by ongoing self-comparisons with their interlocutor, as well as verbal and non-verbal feedback. Similarly, Macintyre and Gregersen (2022), who examined conversations between cross-cultural dyads, revealed that anxiety stemmed from difficulties in comprehending accents from unfamiliar interlocutors, while decreases in anxiety were attributed to finding common ground or changing to more familiar topics mid-conversation. Finally, Talebzadeh et al. (2020) investigated enjoyment during teacher-student conversations. Results revealed that automatic mimicry (i.e., mirroring an interlocutor’s behavior) was the main mechanism through which enjoyment was transferred from between student and teacher (e.g., facial expressions, posture, movement, and vocalization). An implication of these studies is that emotions often emerge because of a desire to maintain interpersonal harmony.

Gregersen et al. (2014) and Boudreau et al. (2018) both report on investigations that are most relevant to the current study. Gregersen et al. (2014) triangulated idiodynamic ratings of anxiety levels and physiological responses (heartrate) for learners during presentation tasks in a Spanish as a foreign language class. They found that anxiety levels were highly volatile, with momentary fluctuations attributed to the presentation topic, learners’ vocabulary knowledge, the audience’s reaction, and learners’ ongoing evaluation of the task experience. A notable finding was that when learners attempted to memorize presentations word-for-word, they were vulnerable to spikes in anxiety caused by problems recalling specific planned vocabulary. This sometimes led to continually rising anxiety levels, exacerbated by learners’ conscious awareness of their anxious state. Underscoring the significance of pre-task planning strategies, Gregersen et al. (2014) notes that learners who planned general ideas but spoke extemporaneously on their topic (i.e., they did not rely on a memorized script) could maintain lower levels of anxiety. Boudreau et al. (2018) conducted an intriguing examination of the rapidly changing relationship between enjoyment and anxiety during a variety of tasks performed by L2 learners. They found that per-person correlations between anxiety and enjoyment (i.e., correlations on individual learner data) varied from highly negative to almost zero, indicating considerable variation between learners. Inspecting intra-individual fluctuation patterns revealed that, on a moment-to-moment timescale, enjoyment and anxiety interacted continuously in complex ways. This dynamic relationship partly depended on task type, with the photo description task engendering both positive and negative emotions as learners’ past experiences depicted in the photos influenced emotions felt during communication. Similar to Gregersen et al. (2014), momentary surges in anxiety were frequently associated with cognitive-linguistic difficulties (e.g., problems retrieving a word from memory).

In sum, idiodynamic studies have focused mostly on anxiety fluctuation patterns in dyadic conversations. They have demonstrated that emotions fluctuate rapidly with corresponding changes in learners’ cognitive processing ability. They also show that anxiety and enjoyment have a complex relationship that is influenced by a wide range of factors, including task factors, pre-task preparation, characteristics of interlocutors, and characteristics of the learners themselves.

### L2 speech fluency

Fluency in L2 task performances can be measured in several ways: *speed fluency* (or speech rate), which is generally measured by the number of syllables of pruned discourse per second (see Ellis and Barkhuizen, 2005; Ch. 7); *repair fluency*, which is operationalized as the frequency of overt repairs or reformulations (see Gotz, 2013); and *breakdown fluency*, as measured by pause frequency (see Saito et al., 2018). Such variables comprise cognitive fluency (Segalowitz, 2010) and reflect the level of efficiency of speech processing mechanisms (i.e., conceptualization, formulation, and articulation) (Levelt, 1989, 1999).

Of interest to this study is breakdown fluency. Higher breakdown fluency can signal a problem related to conceptualization of ideas (Lambert et al., 2021) or a breakdown in the linguistic encoding process (Kormos, 2006; Gotz, 2013). Moreover, breakdown fluency can also vary as a function of the tasks performed (Tavakoli and Skehan, 2005). For example, structured tasks, or tasks that have predictable discourse patterns (e.g., problem-solution tasks, see Leeming et al., 2020; Lambert et al., 2021; Aubrey, 2022; Aubrey and Philpott, 2022; Aubrey et al., 2022) can aid learners in producing more fluent speech (Ahmadian and Tavakoli, 2011; Ahmadian, 2015). These structured tasks might decrease breakdown fluency as they provide learners with multiple “starting points” from which they can gain control over content, which frees attentional resources for conceptualization and formulation processes (Skehan, 2014).

Breakdown fluency might also be closely related to emotion. For example, pausing has been associated with highly stressful moments as a result of momentary “jams” in cognitive processing (MacIntyre and Legatto, 2011; Gregersen et al., 2014; MacIntyre and Serroul, 2015; Boudreau et al., 2018; Macintyre and Gregersen, 2022). Examining fluency more directly, Wood (2016) measured dynamic WTC and fluency in Japanese learners of English and found that, in general, when WTC is high, fluency is high, and when WTC is low, fluency is low. However, the influence of one variable on the other occurred in both directions. That is, fluency sometimes influenced WTC while WTC sometimes influenced fluency. This two-way interaction was similarly observed in Nematizadeh and Wood’s (2019) case study in which L2 learners of Farsi completed a picture description task. They found that high WTC sometimes coincided with frequent pausing when learners had several ideas but hesitated in choosing appropriate lexis and grammar. These studies are important as they shed light on how problems at different stages in speech processing (e.g., idea conceptualization, encoding ideas with language) can lead to breakdown in fluency. It is notable, however, that claims about relationships between emotions and fluency have so far been either speculative (e.g., inferring emotional states via WTC ratings) or qualitative (e.g., inferring emotional states via interviews only). Seeking to advance this line of research, the current study attempts to connect emotion ratings to language production, first quantitatively, using inferential statistics (correlations), and then qualitatively, by exploring reasons for emotional change. Specifically, this research aims to answer the following questions.

1. Based on pausing behavior and idiodynamic ratings, what are the per-person relationships between anxiety, enjoyment, and breakdown fluency during problem-solving task performances?
2. Based on stimulated recall interviews, what factors influence the relationships between anxiety, enjoyment, and breakdown fluency at moments of emotional change during problem-solving task performances?

## Materials and methods

### Participants

Participants included four learners of English who were attending a university in Hong Kong. Based on information from a background questionnaire, all participants spoke Cantonese as their first language (L1) and had scored “4” on the English language subject level of their Hong Kong Diploma of Secondary School Exam (HKDSE), which is benchmarked to the IELTS score range of 6.31–6.51 (Hong Kong Examinations and Assessment Authority, Hong Kong Examinations and Assessment Authority) and equivalent to the Common European Framework of Reference (CEFR) B2/C1 level. Participants were born and raised in Hong Kong and reported having no experience living in an overseas English-speaking country. Despite these common characteristics, there were differences in age, gender, major at university, and use of English, which are summarized in Table 1.

**TABLE 1:** Summary of participant information.

Pseudonym	Gender	Age	Major	Use of English with family or friends	Use of English in the classroom
Isabel	F	19	Hospitality and real estate	Not often	Not often
Diana	F	19	Chinese language education	Never	Never
Brenda	F	22	Chinese language education	Not often	Not often
Travis	M	22	Chinese language education	Sometimes	Sometimes

### Instruments

Participants performed an oral problem-solution task (for the task instructions, see Appendix 1). This task closely followed versions found in recent research (Leeming et al., 2020; Lambert et al., 2021; Aubrey and Philpott, 2022; Aubrey et al., 2022). Learners were required to individually give a 3-min monolog that explained a problem, compared possible solutions, and recommended a solution with reasons. Before the performance, participants were given 10 min to plan for the task by completing a planning worksheet. The worksheet contained the task instructions, the task problem description, and questions to guide learners to understand the situation and problem and to conceptualize their responses and evaluations of their responses (for the planning worksheet, see Appendix 2). The task required participants to consider a problem related to lack of interaction between local and international students on campus that provided the data which was used for this study. In conducting speaking tasks with learners under laboratory conditions similar to the current study, MacIntyre (2019) observed that “an awareness of the camera and researchers” can impact the quality of data collected (p. 6). Thus, to optimize reliability of data collection instruments, a practice task (same task type, different topic) was done with each participant beforehand to orient them to the structure of the desired task performance, familiarize them with the research environment, and train them in the idiodynamic software procedures (see procedures below).

### Procedures

Data were collected during separate sessions with participants. During the task planning stage, the participant made notes on the planning worksheet. The worksheet was collected by the researcher after the 10 min of planning time. A video camera recorded the 3-min speech performance. A timer set for 3 min was placed in front of the participant during the task.

Immediately following the task, the participant watched his/her task performance video and rated their anxiety on a per-second basis using idiodynamic software (for a detailed description of the idiodynamic method, see MacIntyre and Ducker, 2022). The software played the video and simultaneously collected participants’ ratings by recording clicks from a computer mouse. The software displayed the ratings on-screen with colored bars showing increasing or decreasing ratings. The software features an auto-zero function that returned the rating to zero if the mouse was not clicked. The video was then watched a second time to rate enjoyment.

While it is possible that there may be inconsistencies in how different participants used the rating software (Boudreau et al., 2018), learners in this study were provided with feedback on their use of the software during a practice rating session to mitigate such inconsistencies during data collection. During this practice session, learners were also provided with a written definition of anxiety and enjoyment so that they could more intuitively understand what was required from them.

**FIGURE 1:**
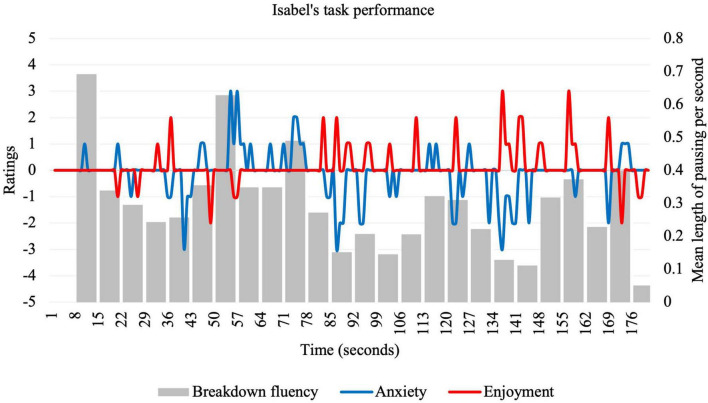
Idiodynamic ratings and breakdown fluency per segment for Isabel’s task performance.

**FIGURE 2:**
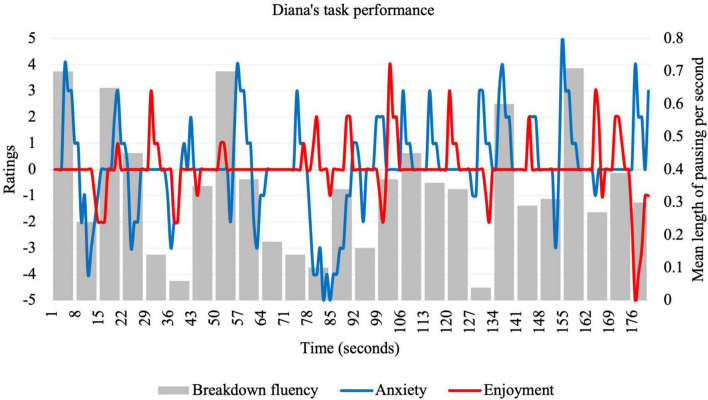
Idiodynamic ratings and breakdown fluency per segment for Diana’s task performance.

**FIGURE 3:**
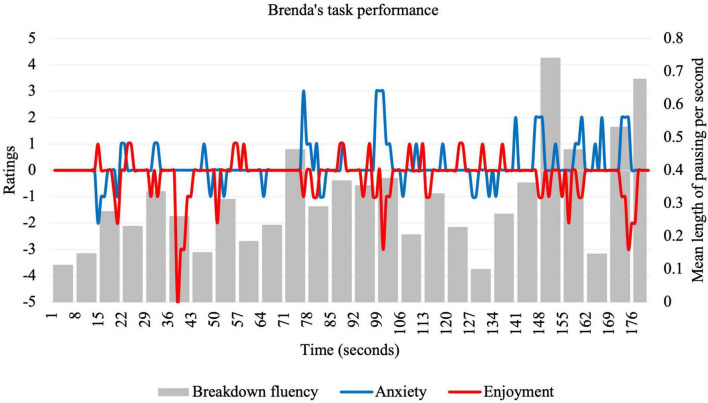
Idiodynamic ratings and breakdown fluency per segment for Brenda’s task performance.

**FIGURE 4:**
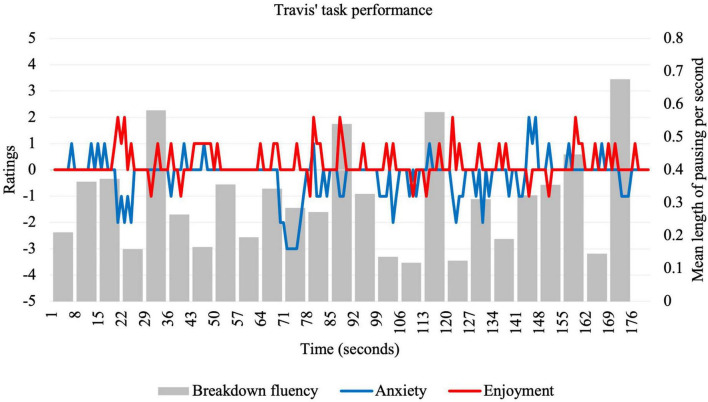
Idiodynamic ratings and breakdown fluency per segment for Travis’s task performance.

After learners completed their ratings, data were then copied to a Microsoft Excel sheet. A graph of the participant’s anxiety and enjoyment ratings were displayed on a computer and shown to the participant to begin the interview (see Figures 1–4). The researcher and the participant looked at the graph together and discussed the trends for each variable across the 3-min period. Examples of questions that were asked by the researcher include: Why did you rate anxiety/enjoyment low at this stage of the task? Can you explain why your ratings remained stable but then increased over this time interval? Why did your ratings for anxiety/enjoyment in the final minutes of the task suddenly decrease? When necessary, the researcher played key segments of the video again for the participant to assist in their recall of specific segments of speech. The participant was also asked to self-evaluate their task performance more generally. A video camera recorded the interviews.

### Analysis

The first aim of the analysis was to produce per-person correlations between enjoyment, anxiety, and breakdown fluency. As an initial step, the 3-min task performances were transcribed by a research assistant (RA) and verified by the researcher. To obtain a measure of breakdown fluency, the task performance sound files were analyzed with PRAAT 6.053 (Boersma and Weenink, 2019) to identify silent pauses of 0.25 s or more (De Jong and Bosker, 2013). The length and position of each of these pauses were added to the transcripts. After initial discussion and practice, interrater reliability was established between the RA who independently coded the transcripts. Pearson product moment correlation coefficients revealed high interrater reliability on the measure of pauses (*r* = 0.921). The transcript was then divided into 25 7-s segments and one 5-s segment (180 s total, or 3 min). This length of segment was chosen because it represented the largest interval of time that any participant produced a run of fluent speech without pausing. This number of data points also satisfied minimum thresholds considered acceptable for inferential statistics (Bonett and Wright, 2000). The mean length of pausing per second for each segment was calculated. Examples of two consecutive segments, with length of pauses (seconds) indicated in parentheses, are provided below:

Example 1: 7-second segment 1 (mean length of pausing per second = 0.69 s):

uh (1.65) vibes (0.65) because (0.56) uh (0.35) we (0.38) we all have (0.74) different cultures (0.51) and

Example 2: 7-s segment 2 (mean length of pausing per second = 0.34 s):

(1.10) we gather and meet each other and many different country people in ah (0.82). the universities (0.45).

Following a precedent for examining idiodynamic ratings in different segments of speech (see MacIntyre and Serroul, 2015; Macintyre and Gregersen, 2022), mean anxiety and enjoyment ratings were calculated for each 7-s speech segment. This was deemed necessary for performing the correlations to answer the first research question. Using the resulting 26 data points, Pearson correlations for each participant were determined between anxiety, enjoyment, and fluency. This approach provides an estimate of the strength of the relationships between task emotions and fluency.

The secondary aim of the analysis was to identify learners’ reasons for patterns of enjoyment and anxiety in the tasks. All interviews were transcribed and examined for specific rationales given for changes in emotions.

## Results

To answer the first research question, measures of anxiety, enjoyment, and breakdown fluency for each individual performance were subjected to correlation analysis. The correlations are shown in Table 2. As can be seen, there were significant positive correlations (strong to moderate) between anxiety and breakdown fluency for three out of the four task performances (Isabel, Brenda, Diana) and one non-significant, very weak correlation (Travis). In contrast, only one task performance produced a significant, moderate correlation between enjoyment and breakdown fluency (Isabel), while others were either non-significant and weak (Brenda), very weak (Travis), and near zero (Diana). Isabel’s task experience produced the only significant and strongly negative correlation between anxiety and enjoyment; others produced non-significant and weak correlations (Brenda, Diana, Travis). Thus, we can see overall positive (anxiety and fluency) and negative relationships (anxiety and enjoyment), but the strength of these correlations varied widely among participants.

**TABLE 2:** Correlations between per-7-s ratings of enjoyment, anxiety and breakdown fluency for each participant.

	Correlation with breakdown fluency		Correlation between anxiety and enjoyment (*r*)
*Participant*	Anxiety (*r*)	Enjoyment (*r*)	
Isabel	0.65**	−0.43*	−0.71**
Diana	0.44*	0.05	−0.27
Brenda	0.51*	−0.38	−0.37
Travis	0.14	−0.13	−0.16

** Is significant at the p < 0.01 level; * is significant at the p < 0.05 level.

To answer research question 2, each individual task experience will now be examined more closely. It should be noted that, during the interviews, participants often repeated reasons for emotional changes; thus, the following interview summary does not tabulate these reasons, but rather provides illustrative examples in full context for each reason given by each participant.

### Isabel’s task performance

Isabel described herself as “not good at speaking fluent English” and claimed that her “ability in grammar and tenses is weak.” However, she felt confident about her task performance, which she attributed to her familiarity with the task topic and opportunity to plan for the task. Among all participants, Isabel’s idiodynamic ratings were second lowest for anxiety (*M* = −0.1; *SD* = 0.85), second highest for enjoyment (*M* = 0.13; *SD* = 0.63), and her breakdown fluency was average (*M* = 0.29; *SD* = 0.16). Her significant (strong to moderate) correlations indicate there are some regular patterns in the way her anxiety, enjoyment and breakdown fluency fluctuate together throughout the task. These patterns are reflected in Figure 1.

Isabel exhibited more frequent breakdowns, higher anxiety, and lower enjoyment to start the task. The period from 49 to 77 s was particularly unenjoyable and anxiety-provoking, with a higher-than-average breakdown fluency. In the corresponding speech segment below, frequent pausing coincided with the use of hesitation devices and false starts:

… accept (0.35) some ah (1.67) cultural (0.89) habits or chara- (0.25) characteristics of different (0.85) people (1.29) For example, cannot- uh (1.68) we are not- (1.16) we will- (1.29) we cannot uh (0.57) accept (0.59) some Indian people having their hands to eat (0.59) because (0.79) we-(0.58) many of us would think that’s dirty (0.46) and maybe we (1.01) don’t.

In explaining this period, Isabel described cognitive problems associated with the struggle to organize and encode ideas with the L2, causing a highly anxious state: “many things come up in my brain at that moment, so I need to organize them again and I feel anxious.” A transition point occurred at the 77–91-s period of the task, in which enjoyment spiked, anxiety dropped to its lowest point, and breakdowns decreased. Isabel attributed this to the problem-solution structure of the task: “at this point, I changed [from describing problems to giving solutions]- I transition successfully… I start giving some solutions more easily.” As she transitioned from the “problem” stage to the “solution” stage, she could draw on new ideas that she was more confident using. This transition seemed to launch her into a period of low anxiety, high enjoyment, and low breakdown fluency that lasted for the remaining task performance. Of particular interest is the period 126–140 s, in which she experienced her highest enjoyment, lowest anxiety, and decreasing breakdown fluency. This portion of the task performance is given below:

… the better solution is that we provide more (0.28) interactive activities so that (0.35) uh (0.59) we can- for example we hold some com (0.32) competition we can gather some different countries (0.31) people to get into a team, (0.30) therefore they will have common language, topic and (0.30) they can have some.

Isabel emphasized that her emotional change in this segment was linked to a surge in confidence from using an idea that she had selected during planning as her recommended solution for the task problem: “I suggest the better solution here… I can imagine how it works when I planned it… it seems more organized.” This example offers some evidence to support Isabel’s general view that planning improved her self-confidence and optimism in expressing herself. Utilizing a pre-planned idea likely lessened cognitive load (i.e., conceptualization) during this portion of the task performances so that Isabel could direct cognitive resources toward producing fluent speech (i.e., formulation and articulation).

### Diana’s task performance

Diana described herself as competent at using English for “simple communication” but said that she often needs to use Google translate. She reported never using English at home. When she uses English with friends, her communication is usually confined to written chat messages using the WhatsApp mobile application. Compared to other participants, Diana’s idiodynamic ratings were average for both anxiety (*M* = 0.11; *SD* = 1.75) and enjoyment (*M* = 0.03; *SD* = 1.00), and exhibited average breakdown fluency (*M* = 0.29; *SD* = 0.19), but variance for each was higher. The more rapid and extreme fluctuations for ratings and breakdown fluency can be seen in Figure 2. For Diana, anxiety tended to sharply rise and fall with similar changes in pausing during the task.

When the interviewer drew Diana’s attention to the several peaks in anxiety, she immediately attributed these to an inability to retrieve lexis from memory: “Yeah um mostly it is I cannot find the right word to say.” As can be seen in Figure 2, these peaks coincide with increases in mean length of pauses, suggesting a problem during the formulation stage of speech production:

A noticeable change in anxiety during the task was the 56–77-s period. She started with a peak anxiety of 4 and ended with the lowest anxiety of the task, −5. This period also coincided with a decrease in average pausing and an increase in enjoyment. Diana pointed out that this was the time when she transitioned from describing the problem to giving a solution, which can be seen in corresponding speech segment below:

… and actually local students are pretty ah (1.12) stubborn (0.36) because (0.86) we (0.25) kind of we can get kind of intimidated by other culture (0.36) So there are some solutions to this problem (0.25). Namely (0.25) firstly (0.40) the university can offer more scholarships so that um (0.36) more university students can (0.35) go on exchange tours and then (0.27) increase their different exposure to other cultures.

A characteristic of Diana’s speech was that breakdown fluency and anxiety were often unrelated to enjoyment. For example, her highest peak in enjoyment was during the 98–106-s period despite also experiencing an increase in pausing. This corresponds to the following speech segment:

… and then ah (1.71) the whole university can be more inclusive to (0.88) different cultures.

Explaining why she felt enjoyment, she described that she planned to talk about cultural inclusivity as a key point, and this was an opportunity to implement her plan: “I was enjoying the speech when I say about cultural inclusive… [I tried] to put inclusive part into my speech here.” However, when asked about why she also experienced an immediate spike in anxiety, she described her mixed feelings about planning:

[Planning] helps reducing of anxiety for me because I have a rough- rough idea of what I should talk about… but also it can restrict like what I say- it can restrict what I want to say because… I cannot say anything else that I want to say.

Thus, it seems that, for Diana, planning led to both self-confidence in using ideas but also a loss of freedom to choose what ideas to express in the moment. Toward the end of the task, Diana commented on the following segment of speech where enjoyment dipped, anxiety spiked but breakdown fluency decreased to its lowest point (126–133 s):

… their experience to share their culture with the local students in a very (0.28) deep way.

Despite occurring during a fluent run of speech, Diana’s negative emotions were aroused by her use of “deep way”—a phrase that she judged to be inappropriate:

I think I was talking about guest speakers coming to give talks about their own cultures in a deep way and… I said “deep way” and I was very very disappointed, so I was less enjoyable.

Even though Diana used the phrase in a fluent segment of speech, she negatively evaluated her language use which aroused an immediate feeling of disappointment.

### Brenda’s task performance

Brenda described herself as proficient at “basic communication” in English but emphasized that she makes grammar mistakes easily. In general, she doesn’t use English with family or friends and uses English only when required in classes.

Compared to other participants, Brenda reported the highest anxiety ratings (*M* = 0.18; *SD* = 0.74), the lowest enjoyment ratings (*M* = −0.19; *SD* = 0.79), and exhibited the highest breakdown fluency (*M* = 0.31; *SD* = 0.16). Similar to Isabel, Brenda’s breakdown fluency significantly correlated with anxiety (positive, weak) but non-significantly correlated with enjoyment (negative, very weak). Fluctuation patters are shown in Figure 3.

Brenda tended to increase her anxiety and breakdown frequency as the task progressed. To prompt Brenda to explain why she ended performance more anxious than she started, she was played the final speech segment (168–180 s) again:

… it won’t be (0.25) too (2.20) too crowded (1.27) and we can leave (2.2) some space for (1.85) students (0.70) to know each other.

Brenda attributed this high anxiety/low enjoyment/high pausing period to cognitive problems (i.e., conceptualizing the idea and struggling to formulate words), which was exacerbated by an awareness of time constraints:

I’m not very fluent [at this time] … when time is running out, my head cannot function… I have a lot of ideas in my mind, but I cannot express it clearly, so I became very nervous. I didn’t enjoy it very much.

An exception to Brenda’s relatively successful start to the task was the 35–42-s segment, in which enjoyment dipped to its lowest point at −5. This period corresponds to the following segment of speech:

… comp (1.31) the wish can come true (0.51) and one of the causes may be that.

Brenda connected her decease in enjoyment to her use of the phrase “wish can come true”:

It’s the sentence “the wish can come true”… I think it’s a silly sentence…. I really hate “the wish can come true” part.

Thus, similar to Diana, Brenda’s negative evaluation of how she used language led to an immediate emotional response that seemingly did not impact her fluency.

### Travis’s task performance

In contrast to other participants, Travis reported that he often speaks to friends and family in English outside of class, and in the interview, expressed a reasonable level of comfort when speaking English. He evaluated his task performance favorably, describing it as “organized” and commending himself on his “time management.” Reflecting his confidence, compared to other participants, Travis exhibited the lowest ratings for anxiety (*M* = −0.22; *SD* = 0.78) and the highest ratings for enjoyment (*M* = 0.21; *SD* = 0.59), with relatively low variance for each. Breakdown fluency during the task (*M* = 0.30; *SD* = 0.16) was comparable to other participants. Figure 4 visually displays fluctuations for the three key variables.

His relatively enjoyable task experience was corroborated by the interview. Travis repeatedly attributed his positive emotions to the personal nature of the topic. In one instance, he explained that he is “currently living with a foreign roommate”—a situation which provided him with ideas he could draw on to use in the task, which generated a spike in enjoyment, decrease in anxiety, and a decrease in breakdown fluency (63–77-s):

… is that (0.49) there’s a policy that can apply in the student residence (0.38) which is to (1.53) um (0.80) ask (0.33) the international students to be allocated with (0.86) one local students.

He explained that the personal nature of this speech segment led to a positive emotional response:

I am totally talking about my personal experience, and I am confident in delivering that- it’s because that’s what we are trying to do in the residence.

Travis also described his appropriate word choices as influencing feelings of enjoyment. He referred to the following speech segment (14–28 s) which coincided with high enjoyment and low anxiety:

… in Hong Kong (1.33) actually so many so called, like, (1.27) multi-cultural university, (0.51) the international students and the local students just couldn’t (0.60) mingle together.

Travis noted that he “used the word ‘mingle.”’ This was significant because it was a word he would not normally use, yet he found it matched his idea well. He rated this moment highly enjoyable as it engendered a sense of satisfaction and pride.

Travis also provided insight into instances where he felt both enjoyment and anxiety simultaneously. Referring to the period 42–49 s, he commented:

I think I am quite satisfied with my delivering of the idea but at the same time I was thinking ‘oh I couldn’t talk about the cultural part’… I planned about saying this….so it gave me the mix of enjoyment and anxiety… I wanted to talk more but I don’t have the time.

Travis thus connected positive emotion to confidently and fluently expressing the idea, but also felt negative emotions associated with regret for not having time to express another planned idea.

The sole instance he felt highly anxious was during the following 142–149-s speech segment:

… just kind of mandatory for them to (1.26**)** to- (1.00**)** to try to understand more about.

His explanation for anxiety echoed other participants’ reasons related to an inability to retrieve lexis: “I think during that part I was hesitant because I couldn’t find the right word.”

A summary of reasons provided by each participant for changes in emotional levels during the task, as well as the corresponding directions of emotion and breakdown fluency change, is given in Table 3.

**TABLE 3:** Perceived factors leading to change in emotions and breakdown fluency during the task.

Reason for change in emotions	Change in anxiety	Change in enjoyment	Change in breakdown fluency
Struggle with formulating language	Isabel + Diana + Travis +	Isabel − Diana − Travis −	Isabel + Diana + Travis +
Struggle with conceptualizing/organizing ideas	Diana + Brenda +	Diana − Brenda −	Diana + Brenda +
Self-evaluating language used	Travis − Diana + Brenda +	Travis + Diana − Brenda −	Travis − Diana − Brenda −
Personalization of task topic	Travis −	Travis +	Travis −
Task time limits	Brenda +	Brenda −	Brenda +
Task structure	Isabel − Diana −	Isabel + Diana +	Isabel − Diana −
Use of ideas/language from task planning	Isabel − Diana ± Travis +	Isabel + Diana + Travis +	Isabel − Diana + Travis −

## Discussion

The first research question asked about the intra-individual relationships between emotions and breakdown fluency. Three out of four learners (Isabel, Diana, Brenda) exhibited significant positive correlations between anxiety and breakdown fluency (see Table 2). This not only suggests that, for most learners, anxiety was debilitating for language performance (Horwitz, 2010; Gregersen and MacIntyre, 2014), but that momentary increases in anxiety tended to lead to momentary increases in length of pauses (and vice versa) during speaking tasks. Compared to anxiety, the relationship between enjoyment and breakdown fluency was weak, as only one learner (Isabel) exhibited a significant negative relationship (see Table 2). Isabel’s feelings of enjoyment might have fostered a more open, less inhibited state (Fredrickson, 2004) that coincided with less hesitant speech. However, for most learners, a diverse set of factors—not directly related to breakdown fluency—may have been behind changes in enjoyment during tasks. Similarly, Isabel was the only learner who exhibited a significant negative correlation between anxiety and enjoyment (see Table 2). This is consistent with the notion that anxiety and enjoyment are independent emotions that do not always change together in a “see-saw fashion” (Dewaele and Pavelescu, 2021), even at a per-second timescale during short communication tasks (Boudreau et al., 2018). The substantial variation in the strength of correlations between learners suggests that, even when learners are exposed to the same task, they each have unique emotional task experiences.

The second research question asked about the factors that influenced the relationship between enjoyment and anxiety. Learners’ reflections were elicited from graphs of anxiety and enjoyment ratings (see Figures 1–4) and centered around significant emotional experiences during the tasks. Reflecting characteristics of a dynamic system (Larsen-Freeman and Cameron, 2008), these factors (see Table 3) were interconnected, rather than distinct, and exerted influence on different timescales. These influences affected learners in different ways, resulting in rapidly fluctuating anxiety and enjoyment, with some unexpected effects on fluency.

Reminiscent of task-based studies that have investigated learners’ affective responses (e.g., Aubrey et al., 2020; Dao and Sato, 2021; Aubrey, 2022), *task-level* factors relate to task design (topic, task structure) and task implementation (time limits, task planning). These factors interacted with individual learner-internal characteristics, such as interests and experiences, to produce emotional change. Travis, for example, drew on his personal life experiences (e.g., living with a foreign roommate) for ideas used in his performance (e.g., suggestions for improving intercultural interaction), providing content to periodically sustain his fluency while also increasing his enjoyment. This reflects findings from previous research that suggests the alignment of task topic and personal experience engenders positive emotions (Lambert et al., 2021, 2022; Dewaele and Pavelescu, 2021; MacIntyre and Wang, 2021; Lambert et al., 2022). Additionally, the problem-solution structure represented an interesting task design influence. Both Isabel and Diana reported the transition from describing the task problem to providing task solutions as a reason for decreasing anxiety and breakdown fluency. This is consistent with previous assertions that tasks with predictable discourse patterns aid learners in producing fluent speech (Ahmadian and Tavakoli, 2011; Ahmadian, 2015). It is interesting to note, however, that for Isabel, the transition from describing the problem to providing solutions seemed to trigger a positive effect that lasted for the duration of the task, but for Diana, the effect was only momentary.

*Task implementation* factors comprised two influences on emotions and fluency: time limitations and pre-task planning. For Brenda, the approaching time limit induced growing anxiety until it became the dominant emotion toward the end of the task. This led to disruptions in cognitive processing and a steady rise in average length of pauses. Thus, rather than causing a momentary spike, anxiety was continually affected (Gregersen et al., 2014; Boudreau et al., 2018). In terms of pre-task planning, three learners (Travis, Isabel, Diana) attributed planning to an increase in enjoyment, and, for two learners, a decrease in breakdown fluency (Travis, Isabel). This might be expected as preparation for a task is likely to increase self-confidence and optimism, creating the conditions for enjoyment. Pre-task planning has also previously been shown to aid in the conceptualization process, leading to greater fluency during the subsequent performance (Skehan and Foster, 1997; Lambert et al., 2021; Aubrey and Philpott, 2022; Aubrey et al., 2022). However, Travis and Diana reported that planning led to surges in anxiety as they felt they had sacrificed chances to speak more extemporaneously on the task topic. Such mixed effects of planning reflect Bui and Teng’s (2018) finding that some learners may prefer not to plan for a task, especially those who are of higher proficiency and familiar with the task topic (as was the case with Travis). This finding also supports Gregersen et al.’s (2014) view that certain pre-task planning strategies, in which learners feel they need to adhere strictly to what they have prepared, render learners more vulnerable to surges in anxiety during performances.

A second category could be described as *cognitive-linguistic* factors (momentary breakdowns in conceptualization and formulation) which relate to lower-level issues that have immediate consequences on speech production processes (Levelt, 1989, 1999). Most participants (Isabel, Diana, Travis) reported how the struggle to remember words led to momentary surges in anxiety, lowered enjoyment, and increased length in pausing, which is indicative of problems in speech processing. This result aligns with previous idiodynamic studies that suggest problems retrieving specific words co-occur with spikes in anxiety (MacIntyre and Legatto, 2011; Gregersen et al., 2014; MacIntyre and Serroul, 2015; Macintyre and Gregersen, 2022) and increased pausing behavior (Wood, 2016; Nematizadeh and Wood, 2019). Such findings provide the clearest evidence for the interconnectedness of cognitive, emotional, and linguistic subsystems, and reinforce the high anxiety/low enjoyment/high breakdown fluency relationship.

A final category could be described as *achievement outcome* factors, which emerged as retrospective emotional responses to language used. According to control-value theory of achievement emotions (Pekrun, 2006), retrospective emotions are outcome-focused emotions experienced when learners evaluate an outcome positively (success) or negatively (failure), which are associated with activating or deactivating effects, respectively. In this study, learners experienced surges in emotions when evaluating language used that they had either planned or placed considerable importance on. For example, when Travis used a word (*mingle*) that he deemed appropriate to explain the problem (*local and international students do not mingle*), he experienced a momentary surge in enjoyment, lowered anxiety, and decrease in breakdown fluency (i.e., the corresponding “activation” response). Such retrospective emotional responses are consistent with flow experiences in which individuals feel a sense of accomplishment that reaffirms they are performing an activity optimally (Nakamura and Csikszentmihalyi, 2002; Aubrey, 2017a,b). In contrast, Diana and Brenda experienced negative retrospective emotional responses when they perceived to have failed in producing an appropriate phrase that was important for expressing their ideas, leading to heightened anxiety and a decrease in enjoyment; interestingly, though, breakdown fluency decreased. This might be explained by the fact that the emotions triggered were low activation (e.g., shame experienced after using inappropriate language) and could be more easily overcome in this context (i.e., in a monolog task with no interlocutor). As retrospective emotions are rarely studied in the context of task performances, more research needs to investigate additional outcome emotions, which would help researchers understand how these moments of reflection energize task behavior.

## Conclusion, limitations, and pedagogical implications

This study has explored the per-person relationships between enjoyment, anxiety, and breakdown fluency, and the factors that learners perceive to influence these relationships at pivotal moments of change during spoken L2 tasks. Significant methodological features of this study include (1) the dynamic measurement of breakdown fluency (pausing behavior) during short segments of speech, and (2) the combined measurement of both enjoyment and anxiety. The findings from this study suggest that anxiety and enjoyment are influenced by task design and implementation factors (which remain relatively fixed during the task) as well as cognitive-linguistic and achievement outcome factors (which arise as task communication unfolds). However, each learner—mediated by their own experiences—responded to these influences in unique ways. Overall, it was observed that surges in anxiety frequently coincided with breakdowns in fluency. In contrast, enjoyment—often manifesting as confidence when using planned ideas, a feeling of achievement from using appropriate language, and positive emotions related to past experiences—had a much weaker relationship with fluency.

Importantly, this research has pedagogical implications for L2 learning and teaching. If teachers want to optimize learners’ fluency during tasks, they should seek ways to reduce anxiety and improve enjoyment during task performances. This research suggests three ways this can be achieved. First, providing learners with a clear task structure can help scaffold their performance. In the case of a monolog task, this might be done by describing to learners the specific stages they need to progress through in their talk (e.g., situation, problem, solution); alternatively, for more advanced learners, teachers might provide them with time to plan their own outline for the task. Second, connecting the task topic to learners’ personal experiences can improve learners’ emotional investment in the task. The teacher can do this by choosing topics that are in line with learners’ interests, or learners might be encouraged to make their own connections to task topics as a preparation activity. Finally, teachers can cultivate positive emotions by highlighting achievement outcomes. After the task is completed, teachers can conduct a post-task reflection activity (e.g., reporting on language they felt they used successfully); during the task, other learners (or the teacher) might be encouraged to use non-verbal gestures (e.g., a nod, thumbs up) to signal to the speaker successful use of language or content.

This study is not without its limitations. First, as this research contains a small sample size and relies on learners’ imperfect ability to express complex thought-processes, results cannot be generalized. Although there is clear value in depicting individual participants’ experiences, there is certainly room for future idiodynamic studies to expand the number of participants to the extent that they might provide generalized findings (for a rare large-scale idiodynamic study, see Ducker, 2021). Second, learners’ anxiety and enjoyment ratings reflect their understanding of these terms, how they interpret their own feelings, and their competency in using the computer software. While learners were given clear definitions of the target emotions and each practiced using the rating software before the task, participants’ ratings should still be interpreted with some caution. Despite these shortcomings, it is hoped that this study provides a more comprehensive picture of emotion and fluency in task performances.

Based on the findings of this study, some suggestions for future research can be made. Pre-task planning is a well-studied implementation variable in the TBLT domain, and considered an important, easy-to-implement condition that reduces learners’ speech processing demands, resulting in more fluent task performances (Willis and Willis, 2012; Pang and Skehan, 2014; Lambert et al., 2021). However, the current study revealed that pre-task planning sometimes provokes surges in anxiety related to feelings of constraint or regret. Future research should employ the idiodynamic method to further explore the impact of different types of pre-task planning on emotions and fluency in task performances. This would add a more nuanced understanding to an established implementation practice. Research should also strive to explore a wider range of emotions in relation to learners’ language production. Selection could be based on the multidimensional model of affect (i.e., dimensions of valence and activation) (Linnenbrink, 2007) or control-value theory (achievement outcome/activity emotions) (Pekrun, 2006). These frameworks may be helpful in predicting which emotions are worthwhile investigating in the context of task-based speaking performances.

## Data availability statement

The raw data supporting the conclusions of this article will be made available by the authors, without undue reservation.

## Ethics statement

The studies involving human participants were reviewed and approved by the Survey and Behavioral Research Ethics Faculty Sub-committee (Faculty of Education), The Chinese University of Hong Kong. The patients/participants provided their written informed consent to participate in this study.

## Author contributions

SA contributed to conception, design of the study, collected the data, performed the analysis, and wrote the manuscript.

## Appendix

**APPENDIX 1:** Task instructions.

A problem related to education in Hong Kong is described below. For this task you must give a 3-min speech in English in response to the problem. In the speech, you should do the following four things in order: (1) explain the problem, (2) compare possible solutions to the problem, (3) recommend one of these solutions, and (4) give reasons for your recommendation. Before giving your speech, you will have 10 min to prepare using a worksheet. After the preparation, the worksheet and problem description will be taken away. Problem: *Having a multicultural university campus does not in itself ensure interaction between international and local students. In fact, at many so-called “multicultural universities” in Hong Kong, international students and local students do not mix and remain isolated in their own social groups. This is a problem because without international-local student interaction on campus, universities are not benefiting from an exchange of ideas, cultures and languages, which is very important to remain globally competitive.*

**APPENDIX 2:** Planning worksheet.

**Pre-Task worksheet** To help you prepare for your 3-min speech, you have 10 min to complete this worksheet. Please write in English. *Part 1.* Answer the questions below. What is the cause of the problem? _________________________________________________________________________________ Who or what is responsible for the problem? _________________________________________________________________________________ How would you describe the problem for someone who has not read about it? _________________________________________________________________________________ Part 2. First, complete table by deciding on four possible solutions to the problem. Then, think about the advantages and disadvantages of each solution and note them in the column. Possible solutions Advantages Disadvantages 1 2 3 Part 3. Which of these solutions do you think is the best? Recommended solution: _____________________________________________________________________ Please decide on 3 reasons for your choice. ___________________________________________________________________________________ ___________________________________________________________________________________ ___________________________________________________________________________________
